# Better Sleep, Better Care: Streamlining Obstructive Sleep Apnea Screening in Psychiatric Outpatients

**DOI:** 10.1192/j.eurpsy.2025.2333

**Published:** 2025-08-26

**Authors:** J. Kim, A. Ramu, D. Khorasani, T. Kainth, A. Javaid, S. Gunturu

**Affiliations:** 1 BronxCare Health System, Bronx, United States

## Abstract

**Introduction:**

Obstructive Sleep Apnea (OSA) significantly complicates psychiatric conditions, yet its systematic screening within psychiatric settings is not common. We present a unique collaborative initiative within a tertiary care psychiatric hospital in South Bronx, USA, with approximately 3000 outpatients. We aim to bridge this gap by implementing a comprehensive OSA screening and referral process to improve patient outcomes.

**Objectives:**

- Implement a standardized Obstructive Sleep Apnea screening in psychiatric outpatient care.
- Enhance collaboration between psychiatry and sleep medicine for integrated care.
- Address compliance barriers by offering at-home sleep diagnostic options.

**Methods:**

Starting in April 2024, patients in the Adult Outpatient Psychiatry Department are being screened for Obstructive Sleep Apnea using the STOP-Bang questionnaire, with this process ending in October 2024. High-risk patients identified through screening will receive WatchPAT Home Sleep Apnea Testing devices, with distribution and testing to be completed by December 2024. Data collected will include the number of patients screened, proportion identified as high-risk, HSAT completion rates, diagnostic outcomes, and subsequent referrals to sleep medicine services.

**Results:**

The STOP-Bang questionnaire has been successfully integrated into routine clinical assessments for psychiatric outpatients, and the screening phase will conclude in October 2024. Preliminary data shows that a substantial number of patients were identified as high risk for Obstructive Sleep Apnea. The distribution of WatchPAT Home Sleep Apnea Testing devices to these high-risk individuals is underway and will be completed by December 2024. Initial results indicate effective triaging of patients and a high rate of compliance with at-home sleep testing. Detailed findings, including the exact number of patients screened, high-risk identification rates, HSAT completion rates, and diagnostic outcomes, will be presented upon project completion.

**Image 1:**

**Figure:**
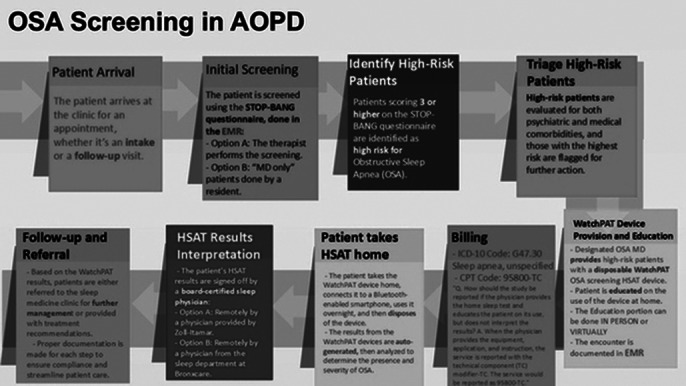

**Conclusions:**

Early findings from this Quality Improvement project suggest that integrating Obstructive Sleep Apnea screening into psychiatric outpatient care is feasible and beneficial. By identifying at-risk patients and providing accessible, at-home diagnostic tools, we aim to enhance patient care and address the underdiagnosed issue of sleep disturbances in psychiatric populations. The project demonstrates the potential for a streamlined, interdisciplinary approach to improve outcomes and set a scalable model for comprehensive patient management in similar settings. Further analysis will focus on the impact of this intervention on psychiatric care and overall patient health outcomes.

**Disclosure of Interest:**

None Declared

